# ﻿Discovery of a new species of the subgenus Japonigekko (Squamata, Gekkonidae, *Gekko*) from the Hengduan Mountains, southwestern China: the best *Japonigekko* mountaineer

**DOI:** 10.3897/zookeys.1215.125043

**Published:** 2024-10-17

**Authors:** Shun Ma, Sheng-Chao Shi, Cheng Shen, Li-Ming Chang, Jian-Ping Jiang

**Affiliations:** 1 Chengdu Institute of Biology, Chinese Academy of Sciences, Chengdu 610041, China Chengdu Institute of Biology, Chinese Academy of Sciences Chengdu China; 2 University of Chinese Academy of Science, Beijing 100049, China University of Chinese Academy of Science Beijing China; 3 Hubei Engineering Research Center for Protection and Utilization of Special Biological Resources in the Hanjiang River Basin, School of Life Science, Jianghan University, Wuhan 430056, China Jianghan University Wuhan China; 4 Mangkang Biodiversity and Ecological Station, Xizang Ecological Safety Monitor Network, Changdu 854500, Xizang, China Mangkang Biodiversity and Ecological Station, Xizang Ecological Safety Monitor Network Changdu China

**Keywords:** *Gekkoalpinus* sp. nov., *Gekkojinjiangensis*, Gekkonidae, molecular phylogeny, morphological characters, new provincial genus record

## Abstract

A new Gekko (subgenus Japonigekko) species, *Gekkoalpinus***sp. nov.**, is described from the Jinsha River Basin in southwestern China, between the border of Mangkang County, Xizang Autonomous Region and Batang County, Sichuan Province, according to the integrative taxonomic results combining molecular data and morphological characters obtained from the type series comprising 11 specimens. Our molecular phylogeny inferred from the mitochondrial *16S* and *ND2* gene fragments indicated that this new species is most closely related to *Gekkojinjiangensis*, but a considerable amount of genetic divergence exists between them (*p*-distance: 3.6%-4.1% (*16S*) and 7.1%–9.1% (*ND2*)). The new species can be distinguished from its congeners via a combination of series morphological characters. The discovery of this new species marks the highest altitudinal range (2400 to 2542 m a.s.l.) recorded for the subgenus Japonigekko and also represents a new provincial record for the genus in Xizang Autonomous Region.

## ﻿Introduction

The gekkonid genus *Gekko* Laurenti, 1768 is widely distributed across eastern and southeastern Asia, Northwest Oceania, and Melanesia. Currently, this genus contains 88 known gecko species and has been divided into seven subgenera: *Archipelagekko*, *Balawangekko*, *Gekko*, *Japonigekko*, *Lomatodactylus*, *Ptychozoon*, and *Rhacogekko* (Wood et al. 2020; Wang et al. 2024; Uetz et al. 2024). Amongst these, *Japonigekko* comprises 33 species, accounting for one-third of the species within the genus. Members of this subgenus are distributed across East Asia, with the majority found in China (20/33), of which the members present the following characters: size moderate; nare usually touching rostral; nasals two or three; dorsal tubercles 0–21 rows; precloacal pores 0–32; postcloacal tubercles 1–4; lateral folds without tubercles (Wood et al. 2020; Hou et al. 2021; Wang et al. 2024; Uetz et al. 2024).

**Figure 1.**
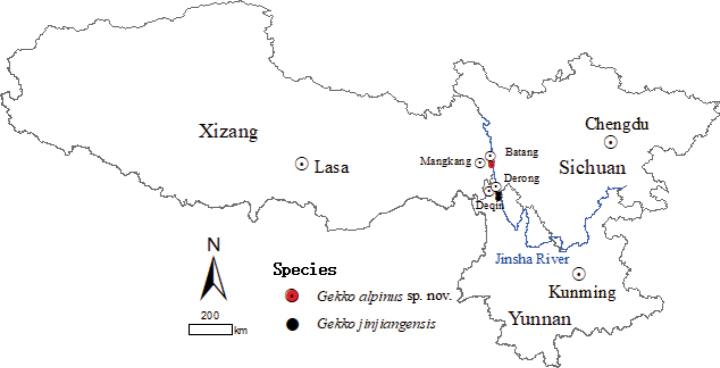
Distribution of *Gekkoalpinus* sp. nov. and its sister taxon *G.jinjiangensis*.

During our field work in the Jinsha River Basin, along the border between Mangkang County of Xizang Autonomous Region and Batang County of Sichuan Province, China, a series of Gekko (Japonigekko) specimens was collected (Fig. 1). This discovery marks the first recorded occurrence of genus *Gekko* in Xizang Autonomous Region (Che et al. 2020). Phylogenetic analysis revealed a significant genetic differentiation between these specimens and their closest relative, *G.jinjiangensis* Hou, Shi, Wang, Jiang & Xie, 2021. Upon closer examination, we found that these specimens are morphologically distinct from *G.jinjiangensis* by having a relatively narrower head, more supralabials and infralabials, more interorbitals and dorsal tubercle rows at the midbody in females, fewer scales in a line from the mental to the front of the cloacal slit, and fewer scale rows at the mid-body. Hence, we describe these specimens as a new species.

## ﻿Materials and methods

### ﻿Specimen preparation

A total of 11 specimens, two specimens (one adult male and one adult female) from Zhubalong Village, Mangkang County, Xizang Autonomous Region collected in July, 2022, and nine specimens (three adult males, four adult females and two subadult females) from Zhubalong Village, Batang County, Sichuan Province in June, 2020 (Fig. 1), were preserved in 75% ethanol and deposited in Chengdu Institute of Biology, Chinese Academy of Sciences (**CIB, CAS**), with their liver tissue samples separately preserved in 95% ethanol for molecular analyses.

### ﻿Molecular data and phylogenetic analysis

Total genomic DNA was extracted by Vazyme FastPure Blood/Cell/Tissue/Bacteria DNA Isolation Mini Kit (Vazyme Biotech Co., Ltd, Nanjing, China) from the liver tissue samples of each specimen. Two mitochondrial gene fragments of partial 16S ribosomal RNA gene (*16S*) and partial NADH dehydrogenase subunit 2 gene (*ND2*) were respectively amplified by primers L3975 (5’-CGCCTGTTTACCAAAAACAT-3’) and H4551 (5’-CCGGTCTGAACTCAGATCACGT-3’) for *16S* (Simon et al. 1994), and rMet-3L (5’-ATACCCCGACAATGTTGG-3’) and rAla-1H (5’-GCCTTAGCTTAATTAAAGTG-3’) for *ND2* (Jonniaux and Kumazawa 2008). The polymerase chain reaction (PCR) was performed in 25 μl reactant with the following cycling conditions: first an initial denaturing step at 95 °C for 5 min; then 35 cycles of denaturing at 95 °C for 40 s, annealing at 53 °C for 40 s and extending at 72 °C for 60 s; last a final extending step at 72 °C for 10 min. PCR products were sequenced by Beijing Qingke New Industry Biotechnology Co., Ltd.

For our phylogenetic analysis, DNA sequences of 49 specimens were used (Table 1), amongst which *ND2* (of specimens No. 1–11, 49) and *16S* (of specimens No. 1–11, 13–17, 44, 49) were sequenced in this study and others were obtained from GenBank. *Gehyramutilata* (Wiegmann, 1834) (No. 49) was used as the outgroup (Pyron et al. 2013). All *16S* (569 bp) and *ND2* (1011 bp) sequences were input in MEGA11 (Tamura et al. 2021) respectively and aligned by MUSCLE (Edgar 2004). Then we calculated the uncorrected pairwise distances (*p*-distance) for each data matrix in MEGA11. Then the concatenation sequences (1580 bp) were prepared for the phylogenetic analysis. The Maximum Likelihood (ML) analysis was performed in IQ-TREE 1.6.12 (Nguyen et al. 2015) based on the best-fit model TIM2+F+I+G4 for the 1^st^ and 3^rd^ codons of *ND2* and all three codons of *16S* and HKY+F+I+G4 for the 2^nd^ codon of *ND2* were computed by ModelFinder for IQ-Tree in PhyloSuite 1.2.3 according to Bayesian Information Criterion (BIC) (Kalyaanamoorthy et al. 2017; Zhang et al. 2020). Ultrafast Bootstrap Approximation (UFB) nodal support was assessed via using ten thousand ultrafast bootstrap replicates and when the value (UFB, %) is ≥ 95, it would be considered as significantly supported (Hoang et al. 2018). The single branch tests were conducted by SH-like approximate likelihood ratio test (SH-aLRT) by 1000 replicates and when the nodal support (SH, %) is ≥ 80, it would also be considered well supported (Stephane et al. 2010). The Bayesian Inference (BI) analysis was conducted via MrBayes 3.2.1 (Ronquist et al. 2012) under the best-fit model GTR+F+I+G4 for the 1^st^ and 3^rd^ codons of *ND2* and all three codons of *16S* and HKY+F+I+G4 for the 2^nd^ codon of *ND2*, which was calculated according to BIC as well by ModelFinder for MrBayes in PhyloSuite 1.2.3. The BI analysis program worked through two independent runs with a four-chain run calculated for 20 million generations using the Markov Chain Monte Carlo (MCMC), sampling every 1000 with the first 25% of samples discarded as burn-in and resulting in a potential scale reduction factor (PSRF) of < 0.005. The nodal support Bayesian posterior probabilities (BI, %) ≥ 95 were considered significantly supported.

**Table 1.** Information and references for *16S* and *ND2* used in this study.

No.	Species	Localities	Voucher ID.	*16S* GenBank Accession No.	*ND2* GenBank Accession No.	Reference
1	*Gekko*alpinus sp. nov.	Mangkang, Xizang, China	CIB 121656	PQ255976	PQ303494	This study
2			CIB 121657	PQ255977	PQ303495	
3		Batang, Sichuan, China	CIB 121658	PQ255978	PQ303496	
4			CIB 121659	PQ255979	PQ303497	
5			CIB 121660	PQ255980	PQ303498	
6			CIB 121661	PQ255981	PQ303499	
7			CIB 121662	PQ255982	PQ303500	
8			CIB 121663	PQ255983	PQ303501	
9			CIB 121664	PQ255984	PQ303502	
10			CIB 121665	PQ255985	PQ303503	
11			CIB 121666	PQ255986	PQ303504	
12	*G.jinjiangensis*	Deqin, Yunan, China	CIB 5334220115	–	MT449431	Hou et al. 2021, this study
13			CIB 5334220088	PQ255987	MT449432	
14			CIB 5334220089	PQ255988	MT449433	
15			CIB 5334220090	PQ255989	MT449434	
16			CIB 5334220100	PQ255990	MT449435	
17			CIB 5334220114	PQ255991	MT449436	
18		Derong, Sichuan, China	CIB 5133380017	–	MT449437	
19			CIB 5133380019	–	MT449438	
20			CIB 5133380021	–	MT449439	
21			CIB 5133380024	–	MT449440	
22			CIB 5133380025	–	MT449441	
23			CIB 5133380026	–	MT449442	
24			CIB 5133380047	–	MT449443	
25	*G.adleri*	Jingxi, Guangxi, China	SYS r001400	MW451654	OR902178	Lyu et al. 2021; Wang et al. 2024
26	*G.auriverrucosus*	Yuncheng, Shanxi, China	NNU Z20050801.004	–	JN019062	Rösler et al. 2011
27	*G.bonkowskii*	Khammouane, Laos	VFU R.2014.10	–	KT266818	Luu et al. 2015
28	*G.chinensis*	Hong Kong, China	SYS r001211	MW451644	OR902183	Lyu et al. 2021; Wang et al. 2024
29	*G.cib*	Chengdu, Sichuan, China	AMB 6567	–	JN019063	Rösler et al. 2011
30	*G.cib*	Hejiang, Sichuan, China	SYS r001489	MW451655	OR902165	Lyu et al. 2021; Wang et al. 2024
31	*G.hokouensis*	Jinzhai, Anhui, China	NNU Z20050902.001	–	JN019060	Rösler et al. 2011
32	*G.hokouensis*	Wuyishan, Fujian, China	SYS r001290	MW451647	OR902173	Lyu et al. 2021; Wang et al. 2024
33	*G.japonicus*	Zhoushan, Zhejiang, China	NNU Z20050801.004	–	JN019059	Rösler et al. 2011
34	*G.japonicus*	Wuyishan, Fujian, China	SYS r000672	MW451628	OR902176	Lyu et al. 2021; Wang et al. 2024
35	*G.khunkhamensis*	Khammouane, Laos	VNUF R.2021.23	–	OL416111	Sitthivong et al. 2021
36	*G.kwangsiensis*	Wuming, Guangxi, China	SYSr 001195	MW451642	OR902175	Lyu et al. 2021; Wang et al. 2024
37	*G.melli*	Dongguan, Guangdong, China	SYS r001742	MW451661	OR902169	Lyu et al. 2021; Wang et al. 2024
38	*G.nadenensis*	Khammouane, Laos	ZFMK 98741	–	KY421618	Luu et al. 2017
39	*G.palmatus*	Zhaoqing, Guangdong, China	SYS r002797	OR903156	OR902179	Wang et al. 2024
40	*G.paucituberculatus*	Baise, Guangxi, China	SYS r002806	OR903154	OR902163	Wang et al. 2024
41	*G.scientiadventura*	Quang Binh, Vietnam	IEBR A.2014.7	–	KP205392	Luu et al. 2014
42	*G.sengchanthavongi*	Khammouane, Laos	VFU R2014.14	–	KT266816	Luu et al. 2015
43	*G.similignum*	Wuzhishan, Hainan, China	SYS r001597	MW451658	OR902185	Lyu et al. 2021; Wang et al. 2024
44	*G.scabridus*	Yanbian, Sichuan, China	CIB YN201909199	PQ255992	MT449429	Hou et al. 2021; this study
45	*G.subpalmatus*	Fenghua, Zhejiang, China	SYS r001762	MW451662	OR902167	Lyu et al. 2021; Wang et al. 2024
46	*G.swinhonis*	Zunhua, Hebei, China	SYS r001814	MW451666	OR902171	Lyu et al. 2021; Wang et al. 2024
47	*G.thakhekensis*	Thakhek, Khammouane, Laos	IEBR A.2014.6	–	KP205396	Luu et al. 2014
48	*G.truongi*	Khanh Hoa, Vietnam	IEBR A.2011.1	–	KP205398	Luu et al. 2014
49	*Gehyramutilata*	Xishuangbanna, Yunnan, China	CIB R201711	PQ255993	PQ303505	this study

### ﻿Morphological comparisons and statistical analysis

Morphological data were obtained from the 11 *Gekkoalpinus* sp. nov. (four males, four adult females, and three subadult females). The terminology and methods of mensural characters and meristic features followed Zhao et al. (1999) and Rösler et al. (2011). Bilateral morphological characters measurements and scale counts were given as left/right.

The mensural characters were measured to the nearest 0.01 mm using a Deli Caliper (DL92150): (1) snout-vent length (**SVL**: from tip of snout to anterior margin of cloaca); (2) tail length (**TaL**: from posterior margin of cloaca to tip of tail); (3) axilla-groin distance (**AGD**: distance between axilla and groin); (4) head length (**HL**: maximum head length from tip of snout to posterior margin of auricular opening); (5) head width (**HW**: maximum head width measured at the angle of the jaws); (6) head height (**HH**: maximum head height from the top of the head posterior to the eyes to the bottom of the lower jaw); (7) snout length (**SL**: from snout tip to anterior corner of eye); (8) eye-ear distance (**EED**: distance between posterior margin of eye to posterior margin of ear opening) (9) maximum eye diameter (**ED**); (10) maximum ear opening diameter (**EOD**); (11) maximum rostral width (**RW**); (12) maximum rostral height (**RH**); (13) maximum mental width (**MW**); (14) maximum mental length (**ML**); (15) forelimb length (**FlL**: length from the base of the palm to the elbow); (16) hindlimb length (**HlL**: distance from the base of heel to the knee).

All mensural characters except for TaL, FIL, and HIL, which were lacking for *G.jinjiangensis*, were statistically analyzed using R v. 4.3.2, and sexes were separated for subsequent comparisons among the samples due to sexual dimorphism within geckos. For analyses, all measurements were ln-transformed to normalize and reduce the variance, and then scaled to remove allometric effects of body size using the following equation: X_a_ = X_ln_–β ∙ (SVL_ln_–SVL_m_), where X_a_ = adjusted value; X_ln_ = ln-transformed measurements; β = unstandardized regression coefficient for each species; SVL_ln_ = ln-transformed SVL; and SVL_m_ = overall average SVL_ln_ of all samples. This project was performed under *GroupStruct* R package (Thorpe 1975, 1976, 1983; Reist 1985; Lleonart et al. 2000; Chan and Grismer 2021). Principal component analysis (PCA) was performed to cluster the morphometrics except SVL, TaL, FIL, HIL related to each species using *prcomp* R function and *factoextra* R package.

The meristic features were taken as the followings: (1) supralabials (**SPL**: number of scales from commissure of jaw to the rostral scale); (2) infralabials (**IFL**: number of scales from commissure of jaw to the mental scale); (3) interorbitals (**IO**: number of scales in a line between anterior corners of eyes); (4) postmentals (**PM**: scales bordering the mental); (5) dorsal tubercles row at midbody (**DTR**); (6) scales in a line from mental to the front of cloacal slit (**SMC**); (7) scale rows at midbody (**SR**); (8) ventral scales at midbody from one ventrolateral fold to the other (**V**); (9) subdigital lamellae under entire first finger (**LF1**); (10) subdigital lamellae under entire fourth finger (**LF4**); (11) subdigital lamellae under entire first toe (**LT1**); (12) subdigital lamellae under entire fourth toe (**LT4**); (13) precloacal pores (**PP**); (14) postcloacal tubercles (**PAT**).

One-way analysis of variance (ANOVA) test was used to evaluate signiﬁcant differences in the mensural and meristic characteristics between the newly collected specimens and *G.jinjiangensis*, with signiﬁcant different variances (*p*-values < 0.05 in the Levene’s test) using the *aov* R function.

Morphological information of *G.jinjiangensis* were obtained from Hou et al. (2021), and for other species, morphological data were taken from the literature (Stejneger 1907; Zhou et al. 1982; Song 1985; Zhao et al. 1999; Goris and Maeda 2004; Rösler et al. 2005, 2011; Toda et al. 2008; Zhou and Wang 2008; Phung and Ziegler 2011; Nguyen et al. 2013; Luu et al. 2014; Ngo et al. 2015; Yang 2015; Luu et al. 2015, 2017; Hou et al. 2021; Lyu et al. 2021; Sitthivong et al. 2021; Zhang et al. 2023; Wang et al. 2024).

## ﻿Results

### ﻿Phylogenetic analysis

**Figure 2.**
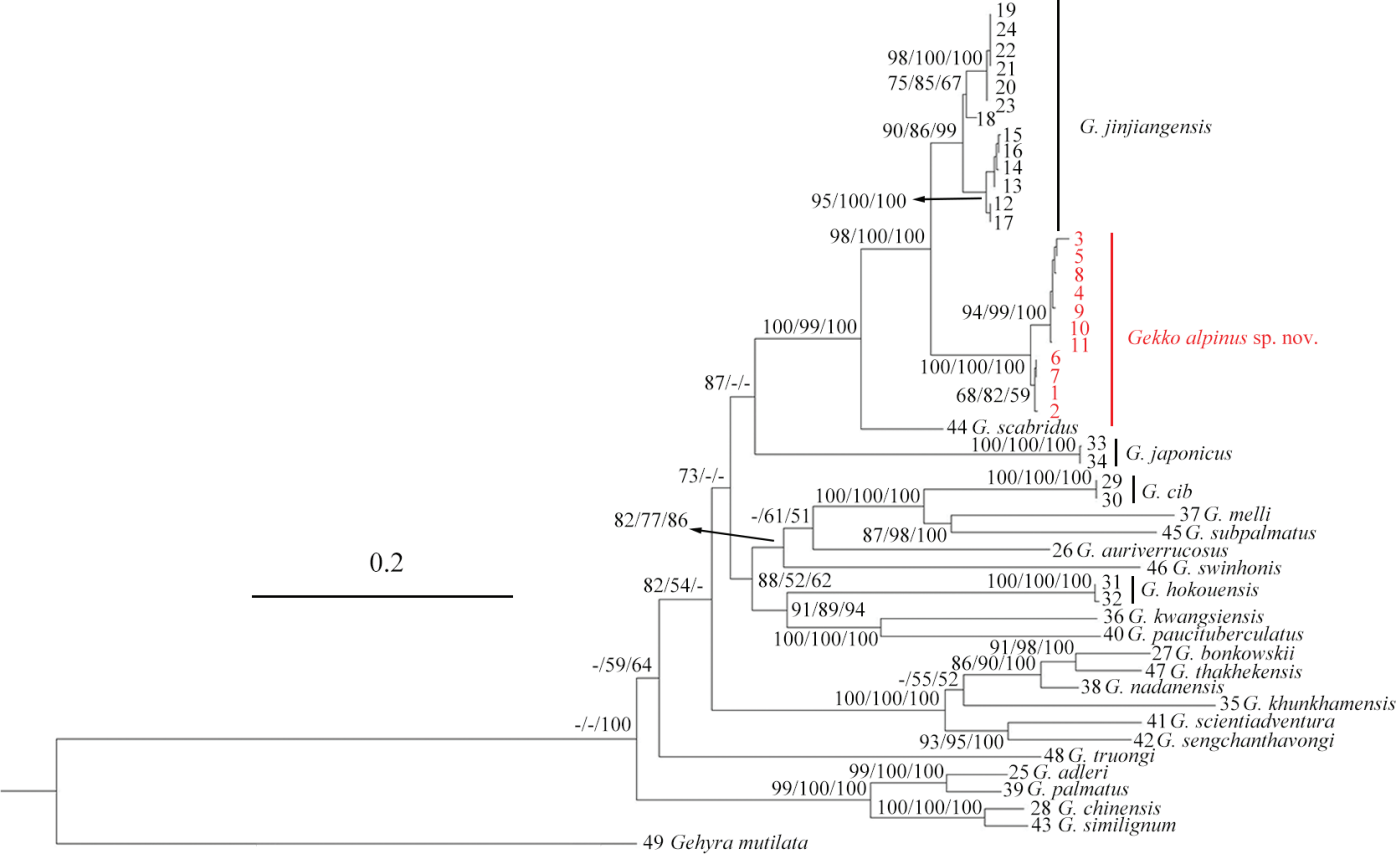
Maximum Likelihood tree topology of *Japonigekko* inferred from the concatenated *16S* and *ND2* gene fragments (1580 bp). The support values of each node present on the tree: SH / UFB / BI (the ones lower than 50 are displayed as “-”). The ID numbers of *Gekkoalpinus* sp. nov. are noted in red.

The new Gekko (Japonigekko) alpinus sp. nov. specimens formed a well-supported sister lineage (SH 100/UFB 100/BI 100) to *G.jinjiangensis* (SH 98/UFB 100/BI 100) with considerable evolutionary differentiation (Fig. 2). The uncorrected pairwise divergences amongst some species of the subgenus Japonigekko studied in this work inferred from the mitochondrial *16S*/*ND2* gene fragments range from 2.2% (*G.chinensis* (Gray, 1842) vs *G.similignum* Smith, 1923) / 5.4% (*G.chinensis* vs *G.similignum*) to 18.4% (*G.chinensis* vs *G.swinhonis* Günther, 1864, and *G.similignum* vs *G.swinhonis*) / 26.5% (*G.melli* (Vogt, 1922) vs *G.similignum*), while the genetic distances amongst *Gekkoalpinus* sp. nov. with its congeners range from 3.6% (vs *G.jinjiangensis*) to 14.0% (vs *G.swinhonis*) for *16S* and 7.1% (vs *G.jinjiangensis*) to 24.1% (vs *G.similignum*) for *ND2* (Tables 2, 3), indicating that *Gekkoalpinus* sp. nov. have distinct interspecific genetic differentiation from its congeners. Based on the molecular results, these *Gekkoalpinus* sp. nov. are supported as representing a new taxon.

**Table 2.** Uncorrected *p*-distance (%) of some species in the subgenus Japonigekko based on the partial mitochondrial *16S* gene sequences. Numbers refer to specimens listed in Table 1.

Species	1–11	13–17	25	28	30	32	34	36	37	39	40	43	44	45
1–11 *Gekko*alpinus sp. nov.	0–0.5													
13–17 *G.jinjiangensis*	3.6–4.1	0–0.6												
25 *G.adleri*	12.6–13.5	12.9–13.3												
28 *G.chinensis*	11.7–12.4	11.4–11.9	7.2											
30 *G.cib*	8.9–9.4	9.2–9.8	13.5	11.9										
32 *G.hokouensis*	11.3–12.2	9.8–10.5	13.6	12.9	13.3									
34 *G.japonicus*	9.8–11.0	9.8–10.5	15.3	14.3	13.7	11.1								
36 *G.kwangsiensis*	11.7–12.8	10.8–11.5	15.6	15.0	11.9	11.9	14.5							
37 *G.melli*	8.9–9.4	9.8–10.4	14.2	13.8	6.0	12.1	13.0	15.0						
39 *G.palmatus*	11.9–12.9	12.5–12.8	3.5	5.0	12.7	14.2	12.8	12.9	10.9					
40 *G.paucituberculatus*	9.8–10.6	9.8–10.5	13.2	12.1	12.0	11.1	13.0	9.8	11.9	14.4				
43 *G.similignum*	11.9–12.7	11.6–12.1	7.4	2.2	12.9	13.6	14.5	16.2	14.2	5.2	13.2			
44 *G.scabridus*	4.7–5.5	4.7–5.4	12.8	12.1	9.7	10.2	10.4	12.4	10.3	11.7	9.7	12.8		
45 *G.subpalmatus*	9.5–9.9	10.9–11.3	16.3	15.9	7.1	13.2	14.1	15.1	10.1	12.8	13.7	16.7	11.3	
46 *G.swinhonis*	13.2–14.0	14.0–14.6	17.2	18.4	11.7	13.4	15.3	17.4	15.3	14.6	14.9	18.4	14.6	15.7

**Table 3.** Uncorrected *p*-distance (%) of some species in the subgenus Japonigekko based on the partial mitochondrial *ND2* gene sequences. Numbers refer to specimens listed in Table 1.

Species	1–11	12–24	25	26	27	28	29–30	31–32	33–34	35	36	37	38	39	40	41	42	43	44	45	46	47	48
1–11 *Gekko*alpinus sp. nov.	0–2.9																						
12–24 *G.jinjiangensis*	7.1–9.1	0–3.5																					
25 *G.adleri*	22.8–23.8	20.3–21.6																					
26 *G.auriverrucosus*	21.2–22.9	20.1–20.8	24.9																				
27 *G.bonkowskii*	18.8–19.4	18.0–19.1	24.6	21.7																			
28 *G.chinensis*	21.8–23.3	20.7–22.2	14.5	24.4	21.2																		
29–30 *G.cib*	22.6–23.7	20.3–20.9	26.3	20.7	19.3	24.2	0																
31–32 *G.hokouensis*	20.7–22.3	18.7–21.1	25.4–25.5	21.5–21.7	20.3	25.0	23.7–23.8	0.3															
33–34 *G.japonicus*	19.8–21.2	17.0–18.1	26.2–26.4	21.0–21.2	19.7–19.9	25.0–25.2	22.8–22.9	22.6–23.0	0.1														
35 *G.khunkhamensis*	21.3–21.7	20.9–21.7	26.0	24.3	15.2	24.6	21.1	24.2	22.6–22.8														
36 *G.kwangsiensis*	22.0–23.5	19.2–19.6	24.3	22.5	21.0	23.2	20.6	21.7–21.8	22.8–23.1	21.5													
37 *G.melli*	23.1–23.6	20.1–21.1	25.3	23.5	21.8	25.3	19.0	24.7	23.8–24.0	23.4	23.8												
38 *G.nadenensis*	18.4–19.4	17.1–18.4	23.3	20.4	6.9	21.2	20.3	21.8–22.1	20.1–20.5	13.8	20.6	21.4											
39 *G.palmatus*	21.4–22.8	21.1–21.6	6.5	23.9	23.6	14.5	26.3	24.6–24.7	25.0–25.2	26.2	23.6	26.1	23.3										
40 *G.paucituberculatus*	20.0–21.7	17.5–18.3	25.5	21.2	19.1	25.6	21.0	21.7	21.6–21.8	21.5	18.9	25.3	18.6	24.6									
41 *G.scientiadventura*	18.4–18.8	17.8–18.6	24.4	20.6	13.9	22.5	20.5	21.4–21.6	21.1–21.2	14.8	21.0	21.6	13.5	23.5	18.6								
42 *G.sengchanthavongi*	19.8–20.3	19.1–19.7	23.8	20.2	14.3	22.1	21.6	22.5–22.9	21.0–21.3	15.7	21.6	22.5	12.0	23.5	18.9	10.5							
43 *G.similignum*	22.8–24.1	21.1–22.6	15.0	25.4	23.1	5.4	25.5	25.1	25.1–25.2	25.0	23.7	26.5	22.7	14.4	25.1	23.1	22.7						
44 *G.scabridus*	11.2–11.8	10.1–10.6	18.5	19.1	19.9	20.3	21.3	17.2	17.2–17.5	21.7	20.1	20.7	19.1	19.2	17.9	17.6	18.4	20.3					
45 *G.subpalmatus*	21.9–23.1	19.2–20.7	25.1	21.8	20.6	24.2	18.0	23.3–23.5	23.3–23.5	22.6	22.3	18.5	18.8	25.6	22.4	20.5	20.6	25.2	20.0				
46 *G.swinhonis*	22.0–23.4	19.4–20.7	25.3	20.8	21.2	25.9	22.0	22.4–22.6	21.9–22.0	23.6	21.8	23.6	21.4	24.5	23.9	22.5	22.1	25.6	19.8	21.7			
47 *G.thakhekensis*	18.8–19.6	18.2–19.5	21.8	20.8	6.8	19.9	20.3	20.8	19.1–19.4	15.9	20.5	20.6	6.8	22.9	18.0	13.1	13.1	21.0	18.9	20.1	23.1		
48 *G.truongi*	20.2–20.6	19.7–20.8	20.5	24.5	22.1	20.8	21.8	22.3	22.0–22.2	22.2	23.1	21.4	22.1	21.8	21.2	22.3	22.0	21.8	18.2	21.6	24.0	20.5	

### ﻿Morphological analysis

Morphological characters of 11 Gekko (Japonigekko) alpinus sp. nov. specimens are presented in Table 4, which can be easily distinguished from all other known congeners (Table 5). By tubercles existing on dorsal body, forelimbs, hindlimbs, and tails, *Gekkoalpinus* sp. nov. can be distinguished from the following 26 species: *G.aaronbaueri* Tri, Thai, Phimvohan, David & Teynié, 2015; *G.adleri* Nguyen, Wang, Yang, Lehmann, Le, Ziegler & Bonkowski, 2013; *G.bonkowskii* Luu, Calame, Nguyen, Le & Ziegler, 2015; *G.canhi* Rösler, Nguyen, Van, Doan, Ho, Nguyen & Ziegler, 2010; *G.chinensis*; *G.cib* Lyu, Lin, Ren, Jiang, Zhang, Qi & Wang, 2021; *G.guishanicus* Lin & Yao, 2016; *G.hokouensis* Pope, 1928; *G.khunkhamensis* Sitthivong, Lo, Nguyen, Ngo, Khotpathoom, Le, Ziegler & Luu, 2021; *G.kwangsiensis* Yang, 2015; *G.liboensis* Zhou, Liu & Li, 1982; *G.melli*; *G.nadenensis* Luu, Nguyen, Le, Bonkowski & Ziegler, 2017; *G.palmatus* Boulenger, 1907; *G.paucituberculatus* Wang, Qi, Zhou & Wang, 2024; *G.scientiadventura* Rösler, Ziegler, Vu, Herrmann & Böhme, 2004; *G.sengchanthavongi* Luu, Calame, Nguyen, Le & Ziegler, 2015; *G.shibatai* Toda, Sengoku, Hikida & Ota, 2008; *G.similignum*; *G.subpalmatus* (Günther, 1864); *G.tawaensis* Okada, 1956; *G.thakhekensis* Luu, Calame, Nguyen, Le, Bonkowski & Ziegler, 2014; *G.truongi* Phung & Ziegler, 2011; *G.vertebralis* Toda, Sengoku, Hikida & Ota, 2008; *G.wenxianensis* Zhou & Wang, 2008; *G.yakuensis* Matsui & Okada, 1968. By having 4–7 precloacal pores in the male, *Gekkoalpinus* sp. nov. differs from *G.kaiyai* Zhang, Wu & Zhang, 2023 (9–12) and *G.scabridus* Liu & Zhou, 1982 (10–15). By having 13–15 subdigital lamellae on fourth toes, *Gekkoalpinus* sp. nov. is different from *G.swinhonis* Günther, 1864 (6–9) and *G.taibaiensis* Song, 1985 (7–8). *Gekkoalpinus* sp. nov. can be differed from *G.japonicus* (Schlegel, 1836) by having fewer interorbitals (IO 22–28 vs 32–35), fewer scale rows at midbody (SR 92–114 vs 130–144) and fewer ventral scales at midbody (32–39 vs 39–44).

**Table 4.** The measurements (in mm) and meristic characters of the type series of *Gekkoalpinus* sp. nov. (“H/P” = holotype and paratype respectively; “F/M” = the gender female and male respectively; “#” = subadult; “*” = the length of regenerated tail; “-” = data unavailable; “+” = tail is broken).

ID	CIB 121663	CIB 121661	CIB 121664	CIB 121658	CIB 121659	CIB 121662	CIB 121666	CIB 121660	CIB 121665	CIB 121656	CIB 121657	Range Mean ± SD
**Type**	H	P	P	P	P	P	P	P	P	P	P	
**Sex**	M	M	M	F	F	F	F	F#	F#	M	F#	
**SVL**	74.16	68.28	65.68	66.76	59.02	66.22	64.34	51.58	50.56	56.44	50.74	50.56–74.16 61.25 ± 7.67
**TaL**	68.02	54.52*	72.06	59.10	59.16*	61.18*	49.22*	59.32	20.28(+)	6.88(+)	39.46*	68.02–72.06 70.04 ± 2.02
**AGD**	27.70	32.34	30.62	34.78	21.16	29.88	31.44	18.62	24.46	24.24	23.72	18.62–34.78 27.18 ± 4.86
**HL**	18.44	17.82	17.22	20.80	16.12	17.42	15.84	15.24	14.92	17.24	19.92	14.92–20.80 17.36 ± 1.76
**HW**	13.26	12.62	12.68	13.24	11.42	12.56	12.56	10.34	10.28	12.22	10.18	10.18–13.26 11.94 ± 1.13
**HH**	6.78	6.74	6.58	5.76	5.14	5.38	5.72	5.12	4.34	7.10	5.44	4.34–7.10 5.83 ± 0.82
**SL**	7.62/7.68	7.12/7.22	6.98/7.16	7.42/7.64	6.72/6.72	7.06/7.16	6.90/6.92	5.82/5.86	5.72/5.74	6.90/6.96	5.78/5.80	5.72–7.68 6.77 ± 0.66
**EED**	5.72/5.66	6.26/6.30	5.62/5.52	5.64/5.72	5.02/5.04	5.78/5.72	5.36/5.46	4.24/4.38	4.64/4.62	5.76/5.76	4.42/4.42	4.24–6.30 5.32 ± 0.61
**ED**	4.36/4.26	3.96/3.98	3.84/3.76	3.94/3.96	3.36/3.38	3.98/3.94	3.26/3.28	2.96/3.02	2.90/2.94	4.24/4.22	3.38/3.40	2.90–4.36 3.65 ± 0.46
**EOD**	1.18/1.12	1.06/1.08	0.74/0.86	1.02/0.98	1.24/1.16	1.14/1.26	0.86/0.88	0.94/0.96	0.86/0.82	1.24/1.24	0.64/0.70	0.64–1.26 1.00 ± 0.18
**RW**	2.12	3.02	2.08	2.52	3.04	2.08	2.92	1.62	1.86	1.56	2.40	1.56–3.04 2.29 ± 0.51
**RH**	1.22	1.42	1.12	1.32	1.44	0.82	1.40	0.84	1.12	1.18	1.18	0.82–1.44 1.19 ± 0.20
**MW**	2.22	2.04	1.66	1.82	1.64	1.42	1.60	1.38	1.86	1.62	1.42	1.38–2.22 1.70 ± 0.25
**ML**	1.62	1.54	1.52	1.48	1.32	1.28	1.52	1.18	1.28	1.82	1.62	1.18–1.82 1.47 ± 0.18
**FIL**	6.86/6.70	7.52/7.34	7.58/7.64	7.58/7.74	6.82/6.88	7.42/7.56	6.42/6.36	5.82/5.98	6.32/6.02	7.98/8.00	6.14/6.16	5.82–8.00 6.95 ± 0.69
**HIL**	8.56/8.48	8.62/8.84	8.72/8.62	8.60/8.58	9.06/9.02	8.72/8.84	7.42/7.64	6.44/6.48	7.34/7.38	7.18/7.22	6.92/7.04	6.44–9.06 7.99 ± 0.85
**SPL**	11/11	10/9	10/10	9/9	10/10	11/12	10/10	10/11	10/10	13/11	10/10	9–13 10.32 ± 0.92
**IFL**	10/10	8/8	9/9	10/10	9/9	9/9	9/9	9/9	.10/9	9/10	9/9	8–10 9.18 ± 0.57
**IO**	27	23	25	24	28	23	28	22	25	24	25	22–28 25.00 ± 2.00
**PM**	2	2	2	2	2	2	2	2	2	2	2	2 2.00 ± 0
**DTR**	15	16	12	17	17	17	15	14	15	17	17	12–17 15.64 ± 1.55
**SMC**	165	175	173	158	162	162	179	163	169	189	181	158–189 170.55 ± 9.25
**SR**	114	109	112	106	101	101	104	101	92	98	95	92–114 103.00 ± 6.54
**V**	33	36	35	34	34	38	36	32	36	38	39	32–39 35.55 ± 2.10
**LF1**	10/10	10/11	10/10	11/11	10/10	11/10	10/9	9/9	9/9	8/9	9/9	8–11 9.45 ± 1.34
**LT1**	11/10	10/10	11/11	10/10	10/10	11/10	10/10	-/9	10/10	9/8	10/9	8–11 9.95 ± 0.72
**LF4**	13/13	13/12	14/13	12/13	14/14	12/13	12/13	13/13	14/12	12/12	13/13	12–14 12.86 ± 0.69
**LT4**	15/14	14/15	14/12	14/14	15/15	14/14	14/14	-/15	15/15	13/14	13/14	12–15 14.14 ± 0.77
**PP**	7	6	4	0	0	0	0	0	0	5	0	4–7 5.50 ± 1.12
**PAT**	1/1	1/1	1/1	2/2	1/1	2/2	1/1	1/1	1/1	2/2	1/1	1–2 1.30 ± 0.46

**Table 5.** Morphological characters of *Japonigekko* (“*” = species distributed in China; “-” = data unavailable; bold = difference between the new species).

No.	Species	SVLmax	SPL	IFL	IO	DTR	SMC	SR	V	LT1	LT4	Web	Fore tubercles	Hind tubercles	Tail tubercles	PP
1*	*Gekkoalpinus* sp. nov.	74.16	9–13	8–10	22–28	12–17	158–189	92–114	32–39	8–11	13–15	0	1	1	1	4–7
2	*G.aaronbaueri*	**80**	13–14	10–11	**34–37**	**0**	–	98–104	**39–43**	**14–17**	14–16	–	**0**	**0**	**0**	3–4
3*	*G.adleri*	75.3	10–15	9–13	**27–36**	**7–11**	168–190	**123–144**	35–44	11–14	11–15	**1**	**0**	1	1	**17–21**
4*	*G.auriverrucosus*	**69**	9–11	9–11	26–27	16–20	–	–	–	6–8	**6–8**	0	1	1	1	**8–11**
5	*G.bonkowskii*	**69.2**	12–14	10–11	**49–50**	**0**	154–169	**117**	37–40	11–13	15	**1**	**0**	**0**	**0**	6
6	*G.canhi*	**99.2**	**14**	10–12	**49–50**	11–12	168–170	**205–227**	**49–51**	**13–16**	14–17	0	**0**	1	**0**	5
7*	*G.chinensis*	72	10–14	9–13	**35–48**	**10**	156–167	**118–140**	37–39	8–10	9–12	**1**	**0**	1	1	**17–27**
8*	*G.cib*	**66.4**	10–12	10–14	**28–36**	**0**	171–196	**128–149**	37–45	9–13	9–17	**1**	**0**	**0**	**0**	7–9
9*	*G.guishanicus*	**64**	–	–	–	**0**	–	–	–	8–10	**8–10**	0	**0**	**0**	**0**	6–8
10*	*G.hokouensis*	70	10–14	8–11	**30–33**	12–18	153–174	**119–130**	36–43	8–11	15–18	0	**0**	**0**	1	5–9
11*	*G.japonicus*	74	9–13	8–13	**32–35**	9–14	169–188	**130–144**	**39–44**	10–12	14–16	0	1	1	1	4–9
12*	*G.jinjiangensis*	**61.6**	7–10	6–9	20–24	12–16	146–169	**111–149**	31–47	8–11	11–15	0	1	1	1	4–5
13*	*G.kaiyai*	**64.99**	9–12	9–13	22–33	11–18	157–209	99–121	30–43	8–9	**7–11**	0	1	1	1	**9–12**
14	*G.khunkhamensis*	75.2	9–10	9–10	**31–32**	**0**	181–185	**127–138**	**42–45**	**13–14**	14–15	**1**	**0**	**0**	**0**	**0**
15*	*G.kwangsiensis*	**69.7**	10–12	**11–13**	**29–31**	**9–11**	**185–208**	**143–156**	**41–45**	11–13	13–18	**1**	**0**	**0**	1	**9–11**
16*	*G.liboensis*	**85**	12	**11**	**40**	**10**	–	–	–	8	**9**	0	**0**	**0**	–	–
17*	*G.melli*	**80.3**	10–13	9–12	**34–40**	**0**	171–192	**148–160**	**44–46**	10–12	11–14	**1**	**0**	**0**	**0**	**9–11**
18	*G.nadenensis*	77.1	12–14	10–12	**28–30**	**0**	175–185	**123–140**	38–40	**13–15**	14–16	**1**	**0**	**0**	**0**	6
19*	*G.palmatus*	79.7	11–15	9–13	27–36	4–12	160–191	**116–147**	36–47	10–13	10–16	**1**	**0**	**0**	1	**23–30**
20*	*G.paucituberculatus*	**85.9**	11	9–10	**37**	**4**	**189–192**	**136–140**	**42–44**	11	11–13	0	**0**	**0**	**0**	**12**
21	*G.scientiadventura*	73	12–14	9–13	**41–51**	**0**	**118–140**	**139–143**	**38–48**	**12–15**	14–17	**1**	**0**	**0**	1	**23–30**
22*	*G.scabridus*	**64**	9–11	9–11	**30**	17–21	–	–	–	6–9	**7–9**	0	1	1	1	**10–15**
23	*G.sengchanthavongi*	77.3	8–10	6–7	**28–32**	0	175–184	**120–135**	35–43	11–14	13–17	**1**	**0**	**0**	**0**	4–5
24	*G.shibatai*	70.9	10–13	10–14	**37–52**	5–14	–	**114–134**	–	–	9–16	0	**0**	**0**	1	**0–3**
25*	*G.similignum*	**58.9**	12–14	**11**	**46–48**	**11**	–	**144–153**	–	11–13	12–14	**1**	**0**	**0**	1	**17**
26*	*G.subpalmatus*	**65.8**	8–12	7–12	**28–37**	**0**	144–190	**129–156**	**39–46**	9–12	11–14	**1**	**0**	**0**	**0**	5–9
27*	*G.swinhonis*	**66**	7–12	7–11	23–24	**6–8**	–	–	**40**	6–9	**6–9**	0	1	1	–	7–9
28*	*G.taibaiensis*	**69**	9–10	8–10	28	–	–	–	–	**6–7**	**7–8**	–	–	–	–	4–6
29	*G.tawaensis*	71	**15**	**13**	–	**0**	–	–	–	10	**12**	0	**0**	**0**	**0**	**0**
30	*G.thakhekensis*	79.2	12–14	10–11	22–26	**0**	165–174	110–116	32–40	11–13	14–15	**1**	**0**	**0**	**0**	1–5
31	*G.truongi*	**95.9**	13–15	**11–13**	**45–48**	**0**	160–172	**131–143**	35–36	11–13	15–17	0	**0**	**0**	**0**	**10–11**
32	*G.vertebralis*	**69.2**	10–15	10–15	**35–50**	2–12	–	**112–139**	–	–	9–17	0	**0**	**0**	**0**	**0–1**
33*	*G.wenxianensis*	59	12	**11**	–	**10**	–	–	**42–44**	**6**	**9**	0	**0**	1	–	6–8
34	*G.yakuensis*	72	12–13	9–13	–	–	–	–	–	10	15	0	**0**	**0**	1	6–8

**Figure 3.**
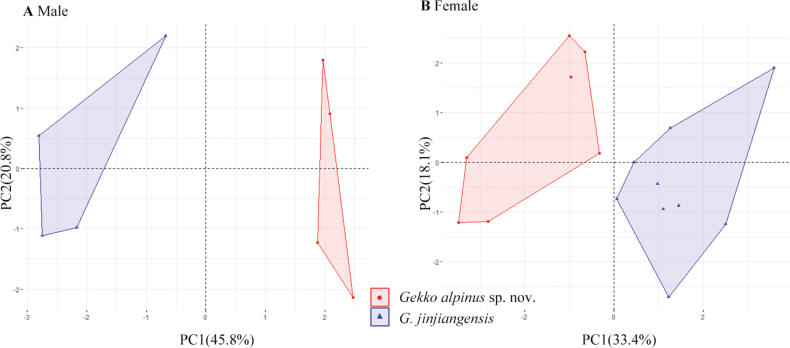
Principal component analysis performed for *Gekkoalpinus* sp. nov. and *G.jinjiangensis* based on 12 commonly used morphological traits (except SVL, Tal, FIL, HIL). Numbers inside the brackets indicate the percentages of the total variance explained by each axis.

The results of the ANOVA indicated that *Gekkoalpinus* sp. nov. is significantly different from its sister taxon *G.jinjiangensis* (Table 6) in the following characters: (1) male: HL, HW, SL, ED, SPL, IFL, SMC, SR, LF4; (2) female: AGD, HL, HW, HH, SL, EOD, SPL, IFL, IO, DTR, SMC, SR, LF4, and LT4. In the PCA analysis (Table 7), the ﬁrst four principal components explained 97.96% of the total variation in the males, where PC1, PC2, PC3, and PC4 eigenvectors accounted for 45.77%, 20.84%, 17.51%, and 13.86% of the total variance respectively, and similarly, the ﬁrst four principal components occupied a considerable proportion in the females, 76.82% of the total, whereas the PC1, PC2, PC3, and PC4 eigenvectors accounted for 33.42%, 18.13%, 15.47%, and 9.80% of the total variance respectively. As illustrated in the scatter plots of PC1 and PC2 (Fig. 3), regardless of sex, the samples of each species cluster together and do not overlap with each other. The results of the ANOVA and PCA indicated that *Gekkoalpinus* sp. nov. was signiﬁcantly different from the closely related *G.jinjiangensis*. As for morphological comparisons, *Gekkoalpinus* sp. nov. can be distinguished from *G.jinjiangensis* by (1) relatively narrower head (HW/HL 0.72 ± 0.01 vs 0.83 ± 0.01 in males and 0.68 ± 0.08 vs 0.81 ± 0.05 in females); (2) more supralabials (SPL 10.63 ± 1.16 vs 8.00 ± 0.50 in males and 10.14 ± 0.74 vs 8.89 ± 0.50 in females) and infralabials (IFL 9.13 ± 0.83 vs 6.63 ± 0.48 in males and 9.21 ± 0.41 vs 7.33 ± 0.70 in females); (3) more interorbitals in females (IO 25 ± 2.14 vs 21.33 ± 1.63); (4) more dorsal tubercles row at midbody in females (DTR 16.00 ± 1.20 vs 13.67 ± 1.15); (5) fewer scales in a line from mental to the front of cloacal slit (SMC 175.50 ± 8.65 vs 155.25 ± 5.63 in males and 167.71 ± 8.34 vs 158.89 ± 6.61 in females); and fewer scale rows at midbody (SR 108.25 ± 6.18 vs 131.75 ± 10.03 in males and 100.00 ± 4.54 vs 123.78 ± 10.20 in females).

**Table 6.** Morphological comparisons of *Gekkoalpinus* sp. nov. with *G.jinjiangensis*. “–” = data unavailable, “*” = *p*-values < 0.05, “**” = *p*-values < 0.01, “***” = *p*-values < 0.001.

	*Gekkoalpinus* sp. nov. *n* = 4	*G.jinjiangensisn* = 4	*p*-values	*Gekkoalpinus* sp. nov. *n* = 7	*G.jinjiangensisn* = 9	*p*-values
	Range Mean ± SD (males)	Range Mean ± SD (males)		Range Mean ± SD (females)	Range Mean ± SD (females)	
**SVL**	56.44–74.16 66.14 ± 6.39	50.2–61.6 56.25 ± 4.26	0.0672	50.56–66.76 58.46 ± 6.90	54.6–61.5 56.57 ± 2.02	0.477
**AGD**	24.24–32.34 28.73 ± 3.08	22.1–24.7 23.83 ± 1.04	0.0547	18.62–34.78 26.29 ± 5.43	20.2–26 24.29 ± 1.65	0.0262*
**HL**	17.22–18.44 17.68 ± 0.50	12.1–15.5 14.03 ± 1.24	0.0000***	14.92–20.80 17.18 ± 2.15	12.2–15.1 13.59 ± 0.89	0.0001***
**HW**	12.22–13.26 12.70 ± 0.37	11.1–12.9 11.65 ± 1.00	0.0047**	10.18–13.24 11.51 ± 1.19	9.2–12.3 11.01 ± 0.87	0.0082**
**HH**	6.58–7.10 6.80 ± 0.19	6.4–7 6.68 ± 0.22	0.144	4.34–5.76 5.27 ± 0.45	4.9–6.8 6.06 ± 0.52	0.0197*
**SL**	6.90–7.68 7.21 ± 0.29	5.4–7 5.98 ± 0.63	0.0012**	5.72–7.64 6.52 ± 0.68	5.2–6.2 5.70 ± 0.41	0.0009***
**EED**	5.52–6.30 5.83 ± 0.26	5.6–6.1 5.40 ± 0.53	0.143	4.24–5.78 5.03 ± 0.55	3.8–5.7 5.03 ± 0.54	0.722
**ED**	3.76–4.36 4.08 ± 0.18	3.3–3.8 3.58 ± 0.19	0.0021**	2.90–3.98 3.41 ± 0.38	3–3.9 3.36 ± 0.27	0.386
**EOD**	0.74–1.24 1.07 ± 0.16	0.6–1.3 0.85 ± 0.29	0.0833	0.64–1.26 0.96 ± 0.18	0.4–1 0.72 ± 0.20	0.0046**
**RW**	1.56–3.02 2.20 ± 0.53	1.7–2.1 1.95 ± 0.17	0.828	1.62–3.04 2.35 ± 0.49	1.9–2.5 2.18 ± 0.17	0.146
**RH**	1.12–1.42 1.24 ± 0.11	1.1–1.2 1.15 ± 0.05	0.339	0.82–1.44 1.16 ± 0.23	1–1.6 1.23 ± 0.20	0.616
**MW**	1.62–2.22 1.89 ± 0.25	1.6–1.9 1.75 ± 0.11	0.631	1.38–1.86 1.59 ± 0.18	1.1–2.1 1.51 ± 0.28	0.319
**ML**	1.52–1.82 1.63 ± 0.12	1.4–1.8 1.68 ± 0.16	0.995	1.18–1.62 1.38 ± 0.15	1.2–2 1.54 ± 0.24	0.202
**FIL**	6.70–8.00 7.45 ± 0.47	–	–	5.82–7.74 6.66 ± 0.65	–	–
**HIL**	7.18–8.84 8.28 ± 0.66	–	–	6.44–9.06 7.82 ± 0.92	–	–
**SPL**	9–13 10.63 ± 1.16	7–10 8.00 ± 0.50	0.0001***	9–12 10.14 ± 0.74	8–10 8.89 ± 0.50	0.0000***
**IFL**	8–10 9.13 ± 0.83	6–7 6.63 ± 0.48	0.0000***	9–10 9.21 ± 0.41	6–9 7.33 ± 0.70	0.0000***
**IO**	23–27 25.00 ± 1.63	21–24 22.50 ± 1.12	0.0803	22–28 25 ± 2.14	20–24 21.33 ± 1.63	0.0027**
**DTR**	12–17 15.00 ± 1.87	13–16 14.25 ± 1.30	0.589	14–17 16.00 ± 1.20	12–15 13.67 ± 1.15	0.0024**
**SMC**	165–189 175.50 ± 8.65	146–161 155.25 ± 5.63	0.0145*	158–181 167.71 ± 8.34	146–169 158.89 ± 6.61	0.0444*
**SR**	98–114 108.25 ± 6.18	124–149 131.75 ± 10.03	0.0136*	92–106 100.00 ± 4.54	111–142 123.78 ± 10.20	0.0001***
**V**	33–38 35.50 ± 1.80	35–47 39.08 ± 4.70	0.14	32–39 35.57 ± 2.26	31–47 38.44 ± 4.52	0.172
**LF1**	8–11 9.75 ± 0.88	8–10 8.85 ± 0.66	0.0901	6–11 9.29 ± 1.53	8–10 8.94 ± 0.66	0.412
**LT1**	8–11 10.00 ± 0.99	8–10 9.31 ± 0.46	0.334	9–11 9.92 ± 0.47	8–10 9.17 ± 0.53	0.0003
**LF4**	12–14 12.75 ± 0.70	10–13 11.92 ± 0.83	0.0154*	12–14 12.93 ± 0.61	11–14 11.94 ± 0.83	0.0020**
**LT4**	12–15 13.88 ± 0.64	12–14 13.23 ± 0.89	0.067	13–15 14.31 ± 0.61	11–14 13.17 ± 0.85	0.0001***
**PP**	4–7 5.50 ± 1.12	4–5 4.50 ± 0.43	0.121	–	–	–
**PAT**	1–2 1.25 ± 0.45	1–2 1.25 ± 0.43	1	1–2 1.29 ± 0.45	1–2 1.11 ± 0.33	0.222

**Table 7.** Variable loadings with the ﬁrst four principal components of *Gekkoalpinus* sp. nov. and *G.jinjiangensis*, with morphometric characters corrected.

Mensural characteristics	Male				Female			
	PC1	PC2	PC3	PC4	PC1	PC2	PC3	PC4
**AGD**	0.2599	-0.4656	-0.0058	-0.0808	-0.1946	0.1391	-0.3604	0.5002
**HL**	0.4186	0.0068	0.0178	0.1257	-0.4724	0.0931	-0.0008	0.1661
**HW**	0.4042	0.1511	0.1207	-0.0109	-0.3806	-0.0953	-0.0450	-0.1095
**HH**	0.1810	-0.1023	-0.0092	0.6899	0.1077	-0.5288	0.2804	0.1429
**SL**	0.3534	-0.2054	0.2237	0.2483	-0.4333	0.0069	0.1578	-0.1221
**EED**	0.3066	0.2964	0.1377	-0.3658	-0.2573	-0.4521	0.3332	0.0167
**ED**	0.4202	0.0147	-0.0935	-0.0302	-0.2948	-0.0683	0.1355	0.2130
**EOD**	0.3339	0.2310	-0.1955	-0.2986	-0.3289	0.3158	0.1295	0.1447
**RW**	-0.0120	-0.3874	-0.4658	-0.3179	-0.2810	-0.3568	-0.2907	-0.0266
**RH**	0.2125	-0.1708	-0.5392	0.0107	-0.0212	-0.4456	-0.4152	-0.2448
**MW**	-0.0851	0.2069	-0.5609	0.3128	-0.1462	0.0650	-0.5343	-0.3276
**ML**	0.0396	0.5866	-0.2162	0.1305	0.1808	-0.2107	-0.2756	0.6636
**Standard deviation**	2.3435	1.5812	1.4494	1.2894	2.0027	1.4749	1.3626	1.0844
**Percentage of total variance**	45.766	20.836	17.506	13.855	33.424	18.128	15.472	9.799
**Cumulative percentage**	45.766	66.602	84.108	97.963	33.424	51.552	67.024	76.823

### ﻿Taxonomic account

#### ﻿ Gekko alpinus sp. nov.

Taxon classificationAnimaliaSquamataGekkonidae

2ABB8647-F0B0-5CD3-8393-19702E112382

https://zoobank.org/CA4785F4-B8D1-4F62-AAF2-B8EB9CC093A3

Figs 4 , 5 , 6 , 7

##### Type materials.

**Figure 4.**
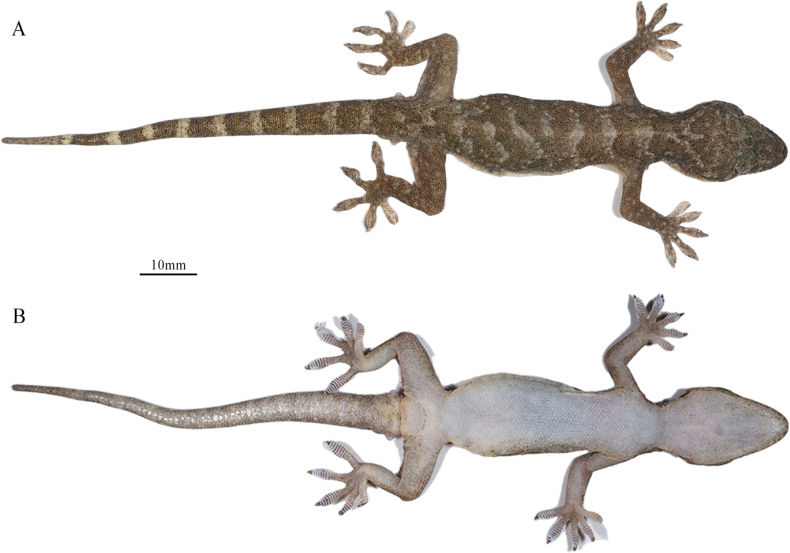
Holotype (CIB 121663, adult male) of *Gekkoalpinus* sp. nov. **A** dorsal view of body **B** ventral view of body. Photographs by S-C Shi.

**Figure 5.**
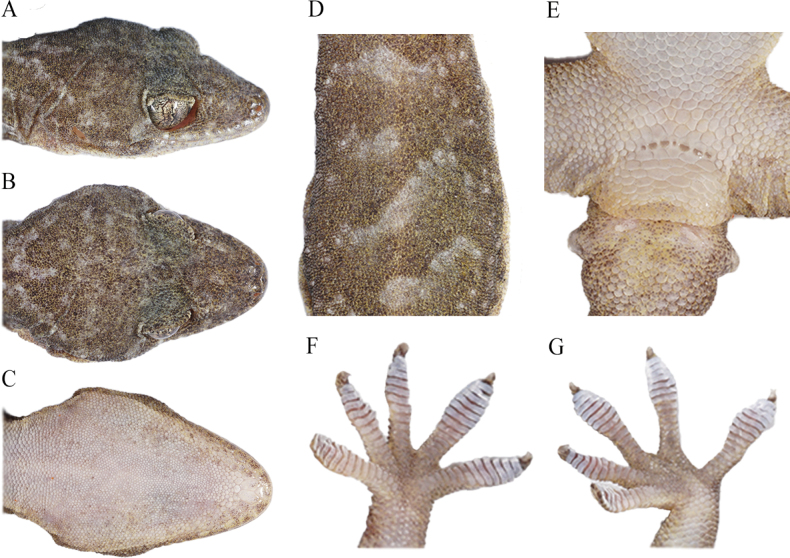
Holotype (CIB 121663, adult male) of *Gekkoalpinus* sp. nov. **A** right lateral view of head **B** dorsal view of head **C** ventral view of body **D** dorsal view of middle body **E** ventral view of precloacal region **F** ventral view of left hand **G** ventral view of left foot. Photographs by S-C Shi.

**Figure 6.**
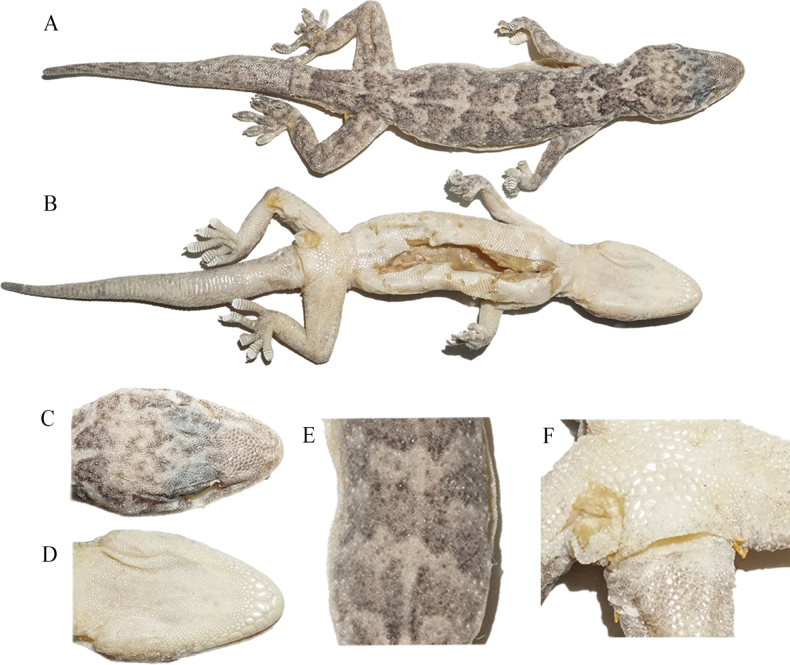
Paratype (CIB 121657, subadult female) of *Gekkoalpinus* sp. nov. **A** dorsal view of body **B** ventral view of body **C** dorsal view of head **D** ventral view of head **E** dorsal view of middle body **F** ventral view of precloacal region. Photos by S Ma.

***Holotype*.** • CIB 121663 (Figs 4, 5), an adult male, collected 26 June 2020 (29.615722°N, 99.02285°E; 2542 m a.s.l.), from Zhubalong Village, Batang County, Ganzi Zang Autonomous Prefecture, Sichuan Province, China by Sheng-Chao Shi, Cheng Shen, Xian-Guang Guo, and Jian-Ping Jiang. ***Paratypes*.** • One adult male: CIB 121661, four adult females: CIB 121658–60, and CIB 121664, and one subadult female: CIB 121662, with the same collection information as the holotype • One adult male: CIB 121665 and one subadult female: CIB QZ088 collected 29 June 2020 (29.731613°N, 99.002355°E; 2494 m a.s.l.), with the same collection locality and collectors’ information • One adult male: CIB 121656 and one subadult female: CIB 121657 (Fig. 6) collected 7 July 2022 (29.758142°N, 99.005975°E; 2400 m a.s.l.), from Zhubalong Village, Mangkang County, Changdu City, Xizang Autonomous Region, China by Cheng Shen, Li-Ming Chang, and Qun-De Zhang.

##### Diagnosis.

(1) body size moderate, SVL 56.44–74.16 mm in adults; (2) head relatively narrow, HW/HL 0.51–0.79; (3) midbody scale rows 92–114, 98–114 in males and 92–106 in females; (4) interorbital scales between anterior corners of the eyes 22–28; (5) ventral scale rows 32–39; (6) tubercles present on dorsal body, forelimbs, hindlimbs and tails; (7) precloacal pores 4–7 in males and absent in the females; (8) subdigital lamellae on first finger 8–11, on fourth finger 12–14, on first toe 8–11, on fourth toe 12–15, no webbing between the fingers and toes; (9) ventral scales between mental and cloacal slit 158–189; (10) nares in contact with rostral; (11) postcloacal tubercles one or two; (12) dorsal surface of body with six or seven large dark taupe bands between nape and sacrum.

##### Description of holotype.

(Figs 3, 4) An adult male, moderate size, SVL 74.16 mm; body slender and trunk relatively elongate (AGD/SVL 0.37); tail little broken at end, TaL 68.02 mm, slightly shorter than SVL.

Head depressed (HH/HL 0.37), length longer than width (HL/HW), distinct from neck. Snout rounded at top, elongate (SL/HL 0.41/0.42), larger than eye (SL/ED 1.75/1.80); rostral irregular polygon, wider than high (RW/RH 1.74) and slightly narrower than mental (RW/MW 0.95); rostral groove absent; rostral in contact with nostril, first supralabial and nasorostral; nares oval, touching rostral, first supralabial three nasals (nasorostral, supranasal, postnasal); one small internasal; snout region medially concave; preorbitals 12/12, preorbital region deeply concave; eye large (ED/HL 0.24/0.23), pupil vertical with crenulated margins; interorbital scales between anterior corners of eyes 27; ear opening oval, obliquely oriented, much smaller than eye (EOD/ED = 0.27/0.26); mental pentagon, width more than length (MW/ML = 0.73); two enlarged postmentals, hexagonal, twice as long as wide; postmentals in contact with mental and first infralabials anteriorly and five gular scales posteriorly; supralabials 11/11; infralabials 10/10; tubercles absent on dorsal head, granulars on anteriodorsal head larger than those on posterior.

Dorsal scales on body smooth, round or oval, granular, juxtaposed; dorsal tubercles 3–4 times the size of dorsal scales, smooth, round to oval, convex, surrounded by 8–10 dorsal scales; dorsal tubercles extending from occiput region to base of tail; tubercles in 15 regular rows at midbody; ventrolateral fold weakly developed, without tubercles; ventrals distinctly larger than dorsal scales, smooth, imbricate and largest in middle of belly; ventral scale rows at midbody 33; scale rows around mid-body 114; ventral scales in a row between mental and cloacal slit 165; precloacal scales enlarged, but no enlarged scales on thighs; precloacal pores seven, in a continuous row.

Forelimbs and hindlimbs well developed, tubercles on fore and hind limbs are present, moderately long, slender; forearm and tibia moderately long, forearm shorter than tibia; digits moderately expanded, both first finger and first toe, clawless, others remaining digits clawed; webbing on fingers and toes absent; subdigital lamellae unnotched and undivided: 10/10-9/10-11/10-13/13-11/11 (manus) and 11/10-11/10-12/12-15/14-13/13 (pes). Relative length of fingers: IV > III > V > II > I; relative length of toes: IV > III > V > II > I.

Tail oval in section, swollen at base, gradually tapering; postcloacal tubercle 1/1, obviously large on tail base side; dorsal scales small, flat, smooth, with dorsal tubercles at the tail base dorsum; ventral scales much larger than dorsal, smooth, and imbricated, with enlarged subcaudal plates arranged into a longitudinal row formed ~ 1/6 TaL distance from the cloaca.

##### Coloration of holotype in life.

(Figs 4, 5). Dorsal surfaces of head, neck and body dark taupe, irregularly scattered with some pale grey threads or blotches, alternatively ornamented with eight large pale grey and seven dark taupe wide bands from neck to the sacrum; an indistinct pale-colored vertebral line is present from the nape to the tail base; dorsal surfaces of limbs, also dark taupe, mottled with small and pale blotches; dorsal tail dark taupe, alternatively ornamented with nine large pale grey and nine larger dark taupe bands, mottled at the ends; ventral skin creamy white, mosaiced with small taupe pigments.

##### Coloration of holotype in preservative.

The coloration pattern of the specimen mostly faded. Dorsal surfaces of head, neck, and body black taupe, but compared to the living status, much wider body area irregularly creamy white, and still alternatively ornamented with eight large creamy white and seven black taupe wide bands from neck to the sacrum. A creamy white vertebral line extends from the nape to the tail terminal; dorsal surfaces of limbs creamy white, mottled with small taupe blotches; dorsal tail dark taupe, alternatively decorated with nine large creamy white and nine larger taupe bands, and mottled towards the end. Ventral skin creamy white, mosaicked with small taupe pigments, though some areas turned creamy yellow due to prolonged alcohol storage.

##### Variation.

All paratypes are very similar to the holotype. Variation of the mensural characters and meristic features among individuals of the type series are presented in Table 4.

##### Distribution and habits.

**Figure 7.**
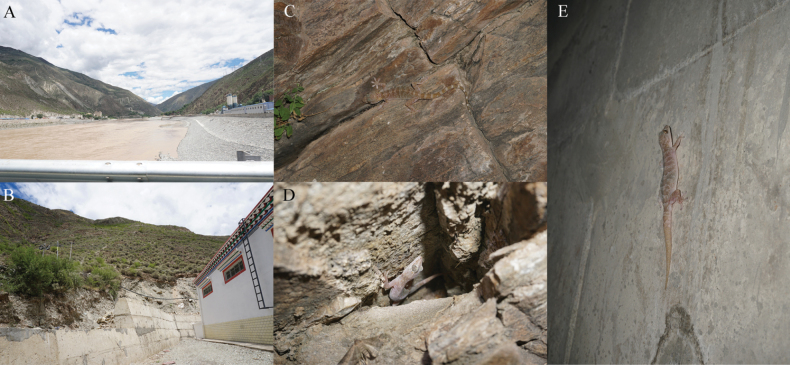
Habitats of *Gekkoalpinus* sp. nov. **A** macrohabitat: Jinsha River dry-hot valley in Zhubalong Village at the border between Batang County, Sichuan Province and Mangkang County, Xizang Autonomous Region **B** microhabitat: house walls **C** one individual found on the dry rocky cliffs **D** one individual found in the rock crevices on cliff **E** one individual found on a house wall. Photos by S-C Shi.

*Gekkoalpinus* sp. nov. is currently known only from the Jinsha River Basin between the border of Mangkang County, Xizang Autonomous Region and Batang County, Sichuan Province, China, at elevations ranging from 2400 to 2542 meters above sea level. This new species is nocturnal and inhabits shrubs or dry rocky cliffs in the arid Jinsha River valley, as well as on building walls (Fig. 7). Ants, discovered in the gut of one specimen, are among the recorded food choices of this species.

##### Etymology.

The specific name *alpinus* is derived from Latin, alpinus, -a, -um, meaning from *Alpēs* (“the Alps”) + -*īnus*, of or pertaining to the Alps, alpine. This refers to the “great high mountains”, referring to not only its distribution range in the great high Hengduan Mountains, but also the highest distribution elevation for all currently known *Japonigekko* species. The suggested common English name is “Alpine Gecko” and the Chinese name is “高山壁虎” (Gāo Shān Bì Hŭ).

## ﻿Discussion

The discovery of *Gekkoalpinus* sp. nov. raises the total species number of the genus *Gekko* to 89, in the subgenus Japonigekko to 34, and within this subgenus in China to 21, including six species distributed in Sichuan Province (*Gekkoalpinus* sp. nov., *G.chinensis*, *G.cib*, *G.japonicus*, *G.jinjiangensis*, and *G.scabridus*). Additionally, this is the only *Gekko* species recorded in Xizang Autonomous Region, marking a new provincial record of this genus in Xizang (Li et al. 2010; Cai et al. 2018; Che et al. 2020; Hou et al. 2021; Lyu et al. 2021).

Hou et al. (2021) reported that the elevation range of *G.jinjiangensis* as 2000 m to 2476 m a.s.l. However, the type series of *G.jinjiangensis* was only found from 2045 m to 2114 m a.s.l. The 2476 m of *G.jinjiangensis* record originally pertains to a *Gekko* population in Batang County, which is actually *Gekkoalpinus* sp. nov., as described in this study. Consequently, we revise the elevation range of *G.jinjiangensis* to 2045–2114 m a.s.l., while *Gekkoalpinus* sp. nov. is distributed between 2400 m to 2542 m a.s.l., making it the highest-altitude *Japonigekko* species currently recognized. Future surveys are recommended to assess the population status of this new species.

The dry-hot valley of Jinsha River in the Hengduan Mountain features habitat heterogeneity and diverse topographic complexity, which supports a variety of reptile species and promotes rapid species evolutionary changes. This is particularly evident in species of *Diploderma* Hallowell, 1861 (Squamata, Agamidae) (Wang et al. 2020; Cai et al. 2022). The discovery of *Gekkoalpinus* sp. nov. also highlights the previously underestimated reptile diversity of this area. The *Gekkoalpinus* sp. nov. populations found on each side of the Jinsha River do not exhibit significant genetic differentiation (uncorrected *p*-distance among No 1, 2, 6, 7 of *16S*/*ND2*: 0–0.2%/0–0.2%) (Tables 2, 3), similar to *Diplodermabatangense* (Li, Deng, Wu & Wang, 2001) (uncorrected *p*-distance of *ND2*: 0–0.4%), implying that the Jinsha River of Hengduan Mountain in Batang and Mangkang do not pose a significant geographical isolation barrier for local reptiles (Wang et al. 2020). To gain a deeper understanding of the reptile diversity patterns and evolutionary histories of Jinsha River Basin in Hengduan Mountain, future field surveys and comprehensive multidimensional analyses are essential.

## Supplementary Material

XML Treatment for Gekko alpinus

## ﻿Additional information

### Author ORCIDs

Shun Ma https://orcid.org/0009-0003-8611-4550

Sheng-Chao Shi https://orcid.org/0000-0003-2337-6572

Li-Ming Chang https://orcid.org/0000-0002-2411-6978

Jian-Ping Jiang https://orcid.org/0000-0002-1051-7797

### Data availability

All of the data that support the findings of this study are available in the main text.
